# Impact of Duodenal Pathology on Oral Drug Bioavailability and Disease Outcomes in Pediatric Crohn’s Disease

**DOI:** 10.3390/ph16030373

**Published:** 2023-02-28

**Authors:** Rebecca Casini, Carrie A. Vyhlidal, Julia M. Bracken, Ashley K. Sherman, Atif Ahmed, Vivekanand Singh, Veronica Williams, Valentina Shakhnovich

**Affiliations:** 1 Department of Pediatrics, North Shore University Health System, Skokie, IL 60201, USA; 2 KCAS Bioanalytical & Biomarker Services, Shawnee, KS 66061, USA; 3 Children’s Mercy Kansas City, Kansas City, MO 64108, USA; 4 Department of Pediatrics, Kansas City School of Medicine, University of Missouri, Kansas City, MO 64018, USA; 5 Department of Pathology, Seattle Children’s Hospitals, Seattle, WA 98105, USA; 6 Department of Pathology, UT Southwestern Medical Center, Dallas, TX 75390, USA

**Keywords:** azathioprine, thiopurine metabolites, absorption, oral drugs, pediatrics, Crohn’s disease, inflammatory bowel disease, IBD, drug disposition

## Abstract

Background: Crohn’s disease with upper gastrointestinal tract involvement occurs more often in children than adults and has the potential to interfere with oral drug absorption. We aimed to compare disease outcomes in children receiving oral azathioprine for the treatment of Crohn’s disease with (DP) and without (NDP) duodenal pathology at diagnosis. Methods: Duodenal villous length, body mass index (BMI), and laboratory studies were compared in DP vs. NDP during the first year post-diagnosis, using parametric/nonparametric tests and regression analysis (SAS v9.4); the data are reported as the median (interquartile range) or the mean ± standard deviation. Thiopurine metabolite concentration (pmol/8 × 10^8^ erythrocytes) 230–400 was considered therapeutic for 6-thioguanine nucleotides (6-TGN), and >5700 was considered hepatotoxic for 6-methylmercaptopurine (6-MMPN). Results: Twenty-six of the fifty-eight children enrolled (29 DP, 29 NDP) started azathioprine for standard medical care, including nine DP and ten NDP who had normal thiopurine methyltransferase activity. Duodenal villous length was significantly shorter in DP vs. NDP (342 ± 153 vs. 460 ± 85 μm; *p* < 0.001) at diagnosis; age, sex, hemoglobin, and BMI were comparable between groups. A trend toward lower 6-TGN was observed in the DP vs. NDP subset receiving azathioprine (164 (117, 271) vs. 272 (187, 331); *p* = 0.15). Compared to NDP, DP received significantly higher azathioprine doses (2.5 (2.3, 2.6) vs. 2.2 (2.0, 2.2) mg/kg/day; *p* = 0.01) and had an increased relative risk of sub-therapeutic 6-TGN. At 9 months post-diagnosis, children with DP had significantly lower hemoglobin (12.5 (11.7, 12.6) vs. 13.1 (12.7, 13.3) g/dL; *p* = 0.01) and BMI z-scores (−0.29 (−0.93, −0.11) vs. 0.88 (0.53, 0.99); *p* = 0.02) than children with NDP. Conclusion: For children with Crohn’s disease, duodenal pathology, marked by villous blunting, increased the risk of sub-therapeutic 6-TGN levels, despite higher azathioprine dosing during the first year post-diagnosis. Lower hemoglobin and BMI z-scores at 9 months post-diagnosis suggest the impaired absorption/bioavailability of nutrients, as well as oral drugs, in children with duodenal disease.

## 1. Introduction

Patients with Crohn’s disease can exhibit histopathologic lesions in both the upper and lower gastrointestinal (GI) tract. Once thought to be a rarity, upper GI tract manifestations of Crohn’s disease, including gastritis and duodenitis, have been reported in 33% of children and 26% of adults, respectively [1]. Among pediatric patients with Crohn’s disease, the prevalence of duodenitis is reportedly higher than in the adult population [2,3,4,5]. A query of retrospective data from our institution (n = 577; 2002–2010) suggests that nearly 50% of children with Crohn’s disease have duodenal pathology at diagnosis [6]. This is important because duodenal pathology can further impair growth for children with inflammatory bowel disease (IBD) by limiting the absorptive capacity for vitamins, minerals, and nutrients [7].

The clinical impact of duodenal pathology on the absorption of oral drugs prescribed to patients with Crohn’s disease (e.g., azathioprine) and their bioavailability is largely unknown. However, evidence is accumulating in support of the altered expression and function of drug-metabolizing enzymes and transporters important to drug disposition in the presence of small bowel inflammation and/or villous blunting from celiac disease [8,9] and Crohn’s disease [10,11,12].

Azathioprine (AZA), an oral immunomodulator used for the treatment of Crohn’s disease, depends on both hepatic and intestinal biotransformation for its efficacy. It is a thiopurine prodrug that is converted to clinically relevant, active, cytotoxic metabolites, 6-thioguanine nucleotides (6-TGN), through a multi-step enzymatic process. Two major enzymatic pathways compete with the activation of AZA. The first is methylation by thiopurine methyltransferase (TPMT) in erythrocytes and the liver to form a therapeutically inactive, hepatotoxic metabolite, 6-methylmercaptopurine (6-MMPN). The other is oxidation by xanthine oxidase/dehydrogenase (XOD) in the small intestine and liver to form 6-thiouric acid (6-TU) [13]. XOD activity in the small intestine is largely responsible for the first-pass metabolism of AZA. The fraction of AZA that escapes XOD metabolism in the small intestine becomes absorbed and available for systemic circulation, where TPMT activity, determined by the TPMT genotype, drives subsequent drug metabolism [13]. The aim of this pediatric study was to assess the impact of duodenal pathology on AZA bioavailability and disease outcomes during the first year of treatment for Crohn’s disease.

## 2. Results

### 2.1. Entire Cohort

Fifty-eight participants (64% male, 83% white) were enrolled in this investigation over the course of 3 years. The majority of patients (66%) presented for diagnostic evaluation within 6 months of initial symptom onset and denied extra-intestinal manifestations of inflammatory bowel disease (76%). The mean age at the time of diagnosis was 13 ± 3.3 years and additional patient demographics are summarized in Table 1.

**Figure 1:**
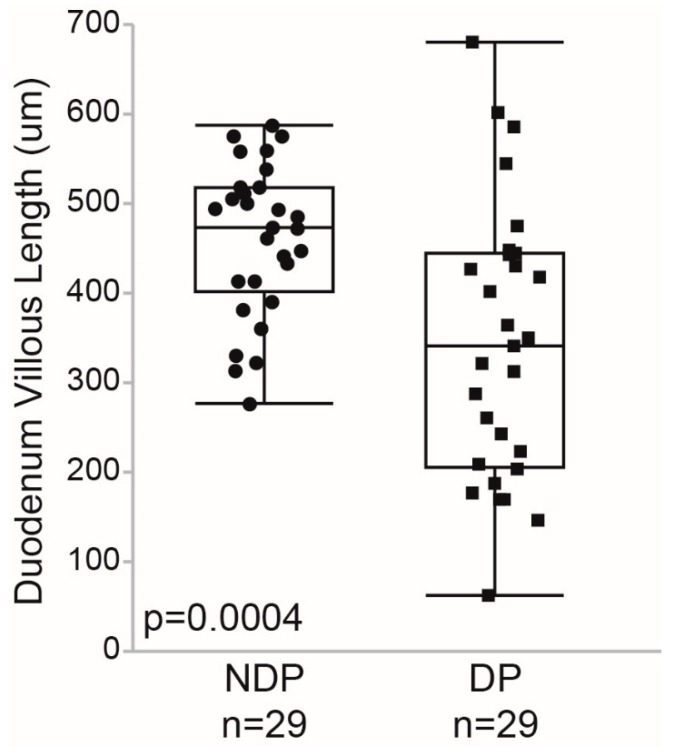
Duodenal villous length for children with Crohn’s disease with duodenal pathology (DP) vs. without duodenal pathology (NDP) at diagnosis.

Crohn’s disease characterized by DP was present in 29 (50%) of the participants and duodenal villous length (μm) was significantly shorter in patients with DP than NDP (342 ± 153 vs. 460 ± 85 μm, *p* = 0.0004; Figure 1).

No statistically significant differences in BMI or laboratory studies were noted between children with DP and NDP at diagnosis, or at 3-month intervals over the course of one-year post-diagnosis (Table 2).

When these comparisons were repeated in all patients initially treated with only oral vs. parenteral medications (i.e., biologicals), no differences were observed between DP and NDP.

### 2.2. Subset of TPMT Normal Metabolizers

**Figure 2:**
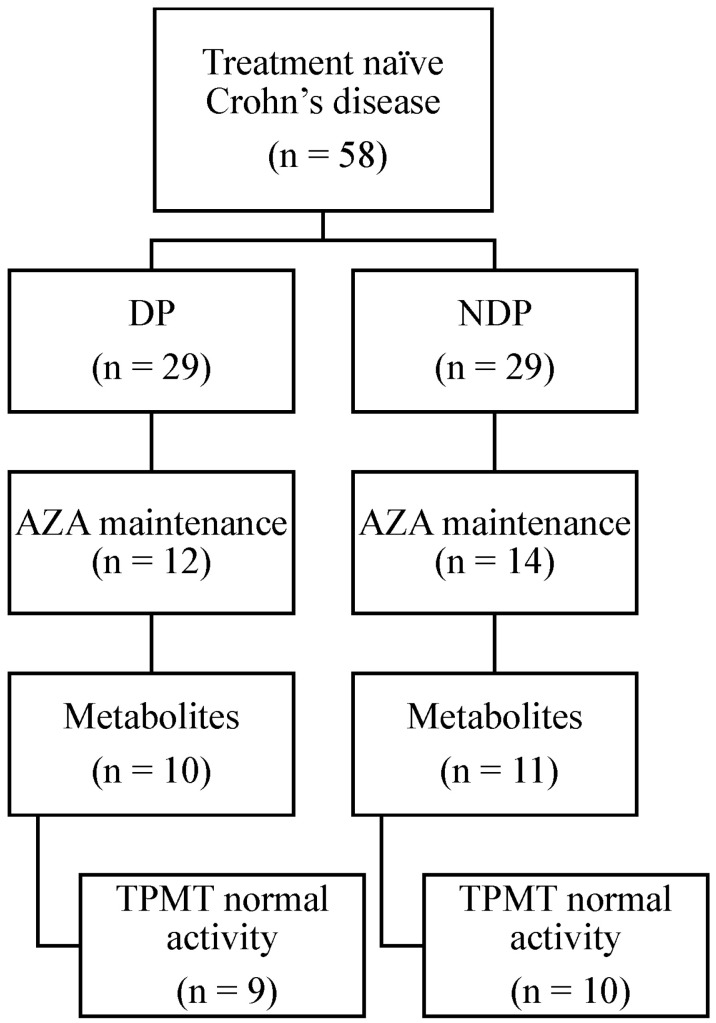
Study participants were divided into 2 groups, based on the presence of duodenal pathology (DP) or no duodenal pathology (NDP) at diagnosis, and further subdivided based on the clinical decision to prescribe maintenance azathioprine (AZA) therapy; TPMT—thiopurine methyltransferase, a polymorphically expressed drug-metabolizing enzyme important to AZA metabolism.

For the 45% of participants who started oral AZA within 6 weeks of diagnosis, 19 had the TPMT normal metabolizer phenotype and thiopurine metabolites available within the first 9 months of initiating AZA treatment (Figure 2).

Patient demographics for the TPMT normal metabolizer cohort are summarized in Table 1. At diagnosis, the DP (n = 9) and NDP (n = 10) subgroups in this cohort did not have statistically significant differences in age, BMI, or laboratory values except ESR, which was higher in DP vs. NDP at diagnosis (*p* = 0.03, Supplemental Table S1), but did not meet our Bonferroni adjusted threshold for statistical significance at α = 0.01. By 3 months of therapy, no statistically significant differences in ESR, using a 0.01 threshold, were observed between groups (9 (8, 17) vs. 10 (9, 16); *p* = 0.70).

The median AZA dose received was higher in DP than NDP (median difference 0.3 mg/kg; *p* = 0.006; Table 3). Two children in the NDP group received AZA doses below the recommended range for TPMT normal metabolizers—1.1 and 1.2 mg/kg, respectively, instead of the recommended 2.0–3.0 mg/kg [14]. Despite DP receiving statistically significantly higher AZA doses, a trend toward lower median 6-TGN (Supplemental Table S1) and higher relative risk of subtherapeutic 6-TGN (Table 3) was noted for DP vs. NDP, although neither reached statistical significance.

While receiving treatment with AZA, children with DP had lower BMI-z scores and Hgb values at 9 months post-diagnosis compared to children with NDP (*p* < 0.03; Table 2). However, only differences in Hgb remained statistically significant after Bonferroni correction for multiple comparisons (*p* < 0.01).

## 3. Discussion

Despite receiving statistically significantly higher AZA doses for the treatment of Crohn’s disease (median difference 0.3 mg/kg/day; *p* < 0.01), children with duodenal pathology were almost twice as likely to have sub-therapeutic 6-TGN levels during the first year of treatment, compared to children without duodenal disease (RR 1.85), although this trend did not reach statistical significance. These children also had a trend toward lower BMI z-scores and hemoglobin at nine months post-diagnosis (Supplemental Table S1), from which we can potentially infer suboptimal disease control with standard AZA dosing, possibly related to the impaired duodenal absorption of oral medications and/or nutrients (e.g., calories for growth, iron for erythrocyte formation). To our knowledge, this is the first study to examine oral drug disposition in children with IBD and duodenal pathology compared to those without duodenal pathology. According to a recently published review, adult data are similarly lacking regarding the fate of orally administered drugs and their bioavailability in the setting of intestinal inflammation in Crohn’s disease [15]. Our findings contribute valuable new pharmacology knowledge that could have implications for the rational drug dose selection of oral medications in the setting of duodenal disease in children with Crohn’s disease. This is important, especially as duodenal disease involvement more often affects children than adults [2,3,4,5], during a critical period of pediatric growth and development.

Although duodenal pathology in newly diagnosed Crohn’s disease in our study was marked by a significant reduction in duodenal villous length, our investigation found no statistically significant differences in growth parameters for children with Crohn’s disease and duodenal pathology vs. no duodenal pathology during the first year post-diagnosis. This is in contrast to some studies that have linked upper tract Crohn’s disease to worse disease outcomes [16]; however, as concluded in a recent review by Kim and Kim, the prognostic implications of upper tract Crohn’s disease involvement remain controversial [17]. Furthermore, the duodenal histopathology available for examination may only be focal, affected by sampling bias, and hence may not truly reflect the impairment of drug absorption or physiologic growth parameters.

Although our sample size is small, the strength of our study resides with the decision to limit AZA treatment subanalysis to patients with a normal TPMT phenotype and documented thiopurine metabolite levels available during the first year of treatment. By controlling for TPMT activity, we attempted to ensure the hepatic and erythrocyte biotransformation of AZA to 6-TGN was comparable among patients with and without duodenal pathology. Therefore, we can infer that the observed differences in circulating 6-TGN concentrations, though not statistically significant due to the small sample size or lower than recommended dosing for two patients in the NDP group, could be due to the upregulation of intestinal XOD activity. Such upregulation of XOD activity was previously observed in celiac disease [9] and is responsible for shunting AZA toward 6-TU formation during first-pass metabolism in the small intestine. Interestingly, an increase in intestinal XOD activity was not observed in a previous study of adults with active ileocolonic Crohn’s disease; however, upper tract disease was not addressed/considered in that investigation [13].

We acknowledge that our findings would be strengthened by measuring 6-TU concentration. However, these metabolite measurements are not routinely obtained for clinical purposes and were, therefore, unavailable for this retrospective study. Another potential limitation of our study is the use of two different assays to measure thiopurine metabolites. We attempted to account for variability in TPMT phenotype designation between assays by grouping TPMT activity > 10.0 EU as normal. Furthermore, the use of two CLIA-approved assays makes our findings more generalizable to the clinical setting, where either assay may be used, based on the institution or third-party payer preference. To the best of our knowledge, children enrolled in this study were adherent to AZA therapy, but we acknowledge that medication adherence may be a confounding factor in data interpretation. Lastly, although differences in ESR observed in TPMT normal metabolizers with DP vs. NDP at diagnosis may be another confounding factor, suggestive of more severe disease in DP than NDP at diagnosis, the difference in ESR did not meet our threshold for significance at α < 0.01. Nevertheless, it is possible that inflammatory burden and/or disease severity could impact AZA biotransformation through enzymatic pathways in the liver or small intestine. Regardless of the mechanism, our data suggest that standard doses of AZA are inadequate for treating Crohn’s disease with duodenal pathology, whether due to a more severe disease phenotype, decreased absorptive capacity in the small intestine secondary to villous blunting, or increased intestinal first-pass metabolism via XOD secondary to inflammation.

Lastly, we acknowledge that the clinical outcomes assessed in our study could have been affected by the inter-individual variability in assessing the duodenal villous blunting observed in the DP study group (Figure 1). Alternatively, clinical outcomes could be confounded by the choice of pharmacotherapy (e.g., oral vs. parenteral administration, immunomodulator vs. biological), differences in drug dose selection, or pharmacogenetics relevant to treatment choice. For this reason, we specifically focused on the subset of children treated with the oral immunomodulator AZA and the TPMT normal metabolizer phenotype. Although our study is limited by sample size, collectively, our findings suggest that the standard dosing of AZA, and perhaps of other oral medications administered to children with IBD, should be increased for patients with duodenal pathology. Alternatively, parenteral drug formulations should be considered. These findings are clinically relevant and important, as based on both our current and retrospective data [6], up to 50% of children with IBD may have duodenal pathology at diagnosis. The incorporation of the relationship between oral drug disposition and the extent of duodenal disease into physiologically based pharmacokinetic models of IBD could provide crucial drug dose individualization knowledge during disease remission and flares. Further investigations are warranted.

To optimize AZA use, future studies should focus on larger, longitudinal patient cohorts, controlling for the TPMT phenotype and employing treat-to-target approaches for therapeutic 6-TGN concentrations to identify the AZA dose increases necessary to achieve therapeutic efficacy in the presence of duodenal pathology, without increasing the risk of hepatic or cytologic toxicity. To isolate the contribution of intestinal XOD to first-pass intestinal metabolism, studies should also capture 6-TU concentrations in children with and without duodenal pathology, and expand to include other oral agents routinely used in the treatment of Crohn’s disease (e.g., iron) in order to better understand the relative contribution of duodenal disease to drug absorption and first-pass metabolism in the small intestine. We believe this knowledge is important for the rational drug dose selection of oral medications administered to children with upper-tract Crohn’s disease during disease flares and potentially for other chronic conditions affecting the small bowel (e.g., celiac disease). Understanding the impact of duodenal pathology on oral drug absorption could also provide relevant information for the development of physiologically based pharmacokinetic models to simulate anticipated differences in drug disposition secondary to duodenal disease and the dose adjustments that may be necessary to negate/override these differences in order to deliver appropriate, individualized treatment.

## 4. Materials and Methods

### 4.1. Study Population

Children with newly diagnosed Crohn’s disease and identified through biorepository activities approved by the institutional review board (IRB) at the Children’s Mercy Hospital (CMH) in Kansas City, MO, were enrolled in this prospective, observational, longitudinal investigation. The study was approved by the CMH IRB and conducted in accordance with the ethical standards of the Declaration of Helsinki. All participants were treatment naïve (i.e., no treatment with immunomodulators, immunosuppressants, biologicals, or mesalamine/sulfasalazine) and had a clinical diagnosis of Crohn’s disease confirmed by a review of the electronic medical record (EMR), as well as by a review of histopathology by two independent pediatric pathologists. Inflammatory bowel disease diagnoses other than Crohn’s disease (e.g., indeterminate colitis, ulcerative colitis) were excluded.

Medical records were queried for growth parameters (body mass index (BMI) and BMI z-score) and laboratory studies (white blood cell count (WBC), hemoglobin (Hgb), albumin (ALB), platelet count (PLT), erythrocyte sedimentation rate (ESR), C-reactive protein (CRP)) at diagnosis and at 3, 6, 9, and 12 months post-diagnosis. All medication use documented in the EMR was reviewed.

### 4.2. Duodenal Histopathology Review

The diagnosis of Crohn’s disease was based on standard-of-care histopathologic, laboratory, and clinical criteria. In addition, duodenal biopsies for all enrolled patients were evaluated by a single independent, experienced pediatric pathologist for the absence, presence, and degree of duodenal inflammation/pathology. The presence of granuloma, chronic active, or non-specific inflammation was used as histopathologic criteria for duodenal pathology (DP study group). Patients without duodenal pathology were assigned to the no duodenal pathology (NDP) study group.

All biopsies were reviewed by a second independent, experienced pediatric pathologist to determine villous length and morphometrics, as previously described by our group [12]. Briefly, three well-oriented villi, in the most inflamed biopsied area, were assessed using commercially available camera software attached to a Nikon microscope (Infinity Analyze, Lumenera Corporation, Ottawa Ontario). Villous length was measured from base to tip and averaged across the three measured villi.

### 4.3. Azathioprine Bioavailability Assessment

All patients prescribed AZA as part of the standard of medical care at disease diagnosis had TPMT enzyme activity, AZA dose (mg/kg/day), and thiopurine metabolite concentrations (6-TGN, 6-MMPN) available in the EMR. The first recorded metabolite concentration in the EMR after AZA initiation was used for statistical analysis. TPMT activity was determined as part of the standard of medical care by either Mayo Medical Labs or Prometheus laboratory testing, depending on third-party payer preference. In order to account for differences in nomenclature between the 2 assays [18,19], TMPT activity reported in Enzymatic Units (EU; also called units/mL red blood cells) was classified as low/deficient if <6.0, intermediate if 6.0–10.0, and normal if >10.0 [18].

### 4.4. Statistical Analyses

Parametric and nonparametric tests, as appropriate, were used to compare clinical variables of interest between children DP and NDP, using SAS v9.4 (SAS Institute Inc., Cary, NC, USA). The statistical significance level was set at α = 0.05, or 0.01 when adjusted for multiple time point comparisons using Bonferroni correction [20]. Duodenal villous length, growth parameters (e.g., BMI), and laboratory values (e.g., ESR, CRP, Hgb, etc.) were compared between study groups via independent sample *t*-tests, reported as the mean and standard deviation, or Wilcoxon Rank Sum tests, reported as the median and interquartile range (IQR). In a subset of patients with the normal TPMT metabolizer phenotype, thiopurine metabolite concentrations after at least 4 weeks of therapy were compared in DP vs. NDP via the Wilcoxon Rank Sum test and Fisher’s Exact test, with results reported as median IQR. 6-TGN concentrations were classified as therapeutic or sub-therapeutic based on the standard-of-care medical care lab reference ranges as follows: 6-TGN 230–400 pmol/8 × 10^8^ erythrocytes therapeutic; < 230 sub-therapeutic. 6-MMPN concentrations were classified as either normal or hepatotoxic based on the standard-of-care medical care lab reference ranges as follows: 6-MMPN < 5700 pmol/8 × 10^8^ erythrocytes normal; >5700 pmol/RBC hepatotoxic.

## Figures and Tables

**Table 1:** Patient demographics at the time of Crohn’s disease diagnosis for all patients (n = 58), and the subset with the normal metabolizer phenotype for thiopurine methyltransferase (TPMT) who received azathioprine dosing based on TPMT phenotype recommendations.

Demographic Information	Duodenal Pathology (n = 29)	No Duodenal Pathology (n = 29)	*p*-Value
Age (years)	12.8 ± 3.4	13.2 ± 3.2	0.700
% Female	41.3%	31.0%	0.412
Ethnicity	79.3% White	86.2% White	0.706
	17.2% Black	6.9% Black	
	3.4% Asian	3.4% Asian	
	0% Hispanic	0% Hispanic	
	0% Multiracial	3.4% Multiracial	
**TPMT Normal Metabolizer Phenotype**	**Duodenal Pathology** **(n = 9)**	**No Duodenal Pathology** **(n = 10)**	***p*-Value**
Age (years)	12.5 ± 2.8	13.1 ± 3.6	0.690
% Female	22.2%	40.0%	0.629
Ethnicity	88.9% White 11.1% Black	100.0% White	0.474

**Table 2:** Clinical outcomes for all patients (n = 58) at diagnosis and at 9 months post-diagnosis.

All	DP (n = 29)	NDP (n = 29)	*p*-Value at Diagnosis	DP (n = 29)	NDP (n = 29)	*p*-Value at 9 Months
BMI z-score	−0.4 ± 1.5	−0.7 ± 1.5	0.46	−0.2 ± 1.2	0.4 ± 1.0	0.13
White blood cells (mcL)	9.1 ± 5.0	8.9 ± 3.0	0.83	7.5 ± 3.1	6.6 ± 1.6	0.29
Hemoglobin (g/dL)	11.0 ± 1.7	11.4 ± 1.9	0.38	12.5 ± 1.0	13.0 ± 1.4	0.27
Platelets (mcL)	425.5 ± 179.8	382.3 ± 142.3	0.31	335.2 ± 102.4	287.4 ± 77.7	0.14
Albumin (g/dL)	3.5 ± 0.7	3.6 ± 0.6	0.35	4.3 ± 0.4	4.3 ± 0.4	0.92
ESR (mm/hr)	37.0 (18, 59)	19.5 (13,44.5)	0.13	13.5 (8.0, 17.0)	10.0 (7.0, 17.5)	0.61
CRP (mg/dL)	2.5 (0.8, 4.2)	3.2 (1.3, 5.5)	0.41	0.6 (0.4, 1.1)	0.6 (0.5, 0.7)	1.0

**Table 3:** Relative risk ratio (RR) and confidence interval (CI) of therapeutic, sub-therapeutic, and hepatotoxic metabolites for TPMT normal metabolizers treated with azathioprine (AZA).

	DP (n = 9)	NDP (n = 10)	Relevant Statistics
AZA dose (mg/kg/day)	2.5 (2.3, 2.6)	2.2 (2.0, 2.2)	*p* = 0.006
6-MMPN	0 hepatotoxic 9 normal	1 hepatotoxic 9 normal	RR = 1.11 (95% CI 0.08, 15.28)
6-TGN	5 sub-therapeutic 4 therapeutic	3 sub-therapeutic 7 therapeutic	RR = 1.85 (95% CI 0.61, 5.63)

## Data Availability

The data are contained within the article.

## Supplementary Materials

The following supporting information can be downloaded at: https://www.mdpi.com/article/10.3390/ph16030373/s1, Supplemental Table S1: Clinical outcomes for subset with the normal TPMT metabolizer phenotype (n = 19) at diagnosis and at 9 months post-diagnosis.
