# Supplementary material for: Impact of Duodenal Pathology on Oral Drug Bioavailability and Disease Outcomes in Pediatric Crohn’s Disease
Source: Pharmaceuticals (Basel). 2023 Feb 28;16(3):373. doi: 10.3390/ph16030373 (PMC10054108; doi:10.3390/ph16030373)
Supplement: Supplementary file 1 [file pharmaceuticals-16-00373-s001.zip › pharmaceuticals-2222451-supplementary (1).pdf]

Supplemental Table S1. Clinical outcomes for subset with the normal TPMT metabolizer phenotype (n=19) at diagnosis and at 9 months post-diagnosis.

TPMT Normal Metabolizer	DP (n= 9)	NDP (n=10)	p-value at diagnosis	DP (n= 9)	NDP (n= 10)	p-value at 9 months
BMI z-score	-0.97±1.5	-0.7±1.1	0.15	-0.6±0.9	0.8±0.9	0.02
White blood cells (mcL)	10.2±6.5	8.1±2.6	0.35	6.6±2.6	5.7±1.0	0.43
Hemoglobin (g/dL)	9.9±1.7	11.4±1.8	0.08	12.1±1.4	13.2±0.8	<0.01*
Platelets (mcL)	472.1±240.5	331.8±102.4	0.11	347.6±126.7	276.8±99.5	0.28
Albumin (g/dL)	3.3±0.6	3.3±0.6	0.91	4.2±0.2	4.1±0.5	0.87
ESR (mm/hr)	37.0 (27.0, 53.0)	16.5 (12.0, 31.0)	0.03	11.0 (7.0, 15.0)	11.0 (10.0, 12.0)	0.94
CRP (mg/dL)	2.5 (0.6, 4.2)	3.6 (1.3, 4.5)	0.59	0.5 (0.4, 1.7)	1.2 (0.6, 2.0)	0.31
6-TGN (units/mL)	–	–	–	164 (117, 271)	272 (187, 331)	0.11
6-MMPN (units/mL)	–	–	–	935 (694, 1262)	890 (711, 2446)	0.90

** Significant after Bonferroni correction for multiple time point comparisons*

Thiopurine methyltransferase (TPMT), duodenal pathology (DP) and no duodenal pathology (NDP) at time of Crohn's disease diagnosis.
